# Incorporating multi-stage diagnosis status to mine associations between genetic risk variants and the multi-modality phenotype network in major depressive disorder

**DOI:** 10.3389/fpsyt.2023.1139451

**Published:** 2023-03-02

**Authors:** Li Zhang, Mengqian Pang, Xiaoyun Liu, Xiaoke Hao, Meiling Wang, Chunming Xie, Zhijun Zhang, Yonggui Yuan, Daoqiang Zhang

**Affiliations:** ^1^College of Computer Science and Technology, Nanjing Forestry University, Nanjing, China; ^2^College of Computer Science and Technology, Nanjing University of Aeronautics and Astronautics, Nanjing, China; ^3^Department of Psychosomatic and Psychiatry, Zhongda Hospital, School of Medicine, Southeast University, Nanjing, China; ^4^School of Artificial Intelligence, Hebei University of Technology, Tianjin, China; ^5^Department of Neurology, ZhongDa Hospital, School of Medicine, Southeast University, Nanjing, China

**Keywords:** imaging genetics, major depressive disorder, multi-modality, multi-stage diagnosis status, single-nucleotide polymorphisms

## Abstract

Depression (major depressive disorder, MDD) is a common and serious medical illness. Globally, it is estimated that 5% of adults suffer from depression. Recently, imaging genetics receives growing attention and become a powerful strategy for discoverying the associations between genetic variants (e.g., single-nucleotide polymorphisms, SNPs) and multi-modality brain imaging data. However, most of the existing MDD imaging genetic research studies conducted by clinicians usually utilize simple statistical analysis methods and only consider single-modality brain imaging, which are limited in the deeper discovery of the mechanistic understanding of MDD. It is therefore imperative to utilize a powerful and efficient technology to fully explore associations between genetic variants and multi-modality brain imaging. In this study, we developed a novel imaging genetic association framework to mine the multi-modality phenotype network between genetic risk variants and multi-stage diagnosis status. Specifically, the multi-modality phenotype network consists of voxel node features and connectivity edge features from structural magnetic resonance imaging (sMRI) and resting-state functional magnetic resonance imaging (rs-fMRI). Thereafter, an association model based on multi-task learning strategy was adopted to fully explore the relationship between the MDD risk SNP and the multi-modality phenotype network. The multi-stage diagnosis status was introduced to further mine the relation among the multiple modalities of different subjects. A multi-modality brain imaging data and genotype data were collected by us from two hospitals. The experimental results not only demonstrate the effectiveness of our proposed method but also identify some consistent and stable brain regions of interest (ROIs) biomarkers from the node and edge features of multi-modality phenotype network. Moreover, four new and potential risk SNPs associated with MDD were discovered.

## 1. Introduction

Major depressive disorder (MDD) is a serious mental illness characterized by persistent sadness, lack of energy, loss of interest, sleep disturbance, and a high risk of suicide, with a high lifetime prevalence of MDD (16.2%) (1). It already affects about 350 million people worldwide and is expected to be the second most debilitating disease in the world by 2030 (2). Patients with major depression are often suicidal and have a risk of suicide that exceeds 10 times that of the general population (3). However, the etiology of MDD is still not clear. The pathogenesis varies for each individual, and an uniform pathogenesis has not been found in the literature. Currently, the diagnosis of depression mainly depends on patients clinical symptoms or the scores of Hamilton Depression Rating Scale (4). Due to human subjectivity, these methods cannot provide an objective diagnosis. Thus, effective prevention and early diagnosis are the important and urgent research topic for MDD.

With the rapid development of neuroimaging technology, magnetic resonance imaging (MRI), functional magnetic resonance imaging (fMRI), diffusion tensor imaging (DTI), and positron emission tomography (PET) are widely used for the prediction and diagnosis of mental illness and neurological disorders (5–7). Among them, resting-state fMRI (rs-fMRI) can reflect the neural activity of the brain and thus becomes the most popular brain imaging technology to discriminate MDD from healthy controls. Many studies have been made with the intent to explore the potential neuroimaging biomarkers for MDD diagnosis based on brain functional connectivity (FC). Yao et al. proposed a temporal adaptive graph conventional network(GCN), which not only took advantage of both spatial and temporal information using resting-state FC patterns and time-series but also explicitly characterized the subject-level specificity of FC patterns (8). Thereafter, Yao et al. made the full use of multiple indexes derived from rs-fMRI and designed a tensor-based multi-index representation learning framework for fMRI-based MDD prediction (9). Kong et al. first developed a spatio-temporal GCN framework to learn discriminative features from FC for automatic diagnosis and antidepressant treatment response prediction for MDD (10). Subsequently, this team successively proposed a novel multi-stage graph fusion networks framework by integrating the multiple stages of data representations, which fully considered the interactions between the white matter and the gray matter (11). Aforementioned diagnosis methods have the ability to capture dynamic FC alterations from rs-fMRI, which are considered as potential neuroimaging biomarkers to aid diagnosis and treatment for MDD. However, these methods focus on single imaging modality (e.g., rs-fMRI) and are limited in discovering the consistent biomarkers across multiple modalities.

In recent years, high-throughput genotyping technology coupled with brain neuroimaging provides a great promise to investigate the role of genetic variation on the brain structure and function and emerges as a new research field, namely, imaging genetics. The major task of this field is to measure the association between genetic variation (e.g., SNP) and neuroimaging biomarkers extracted from different imaging modalities. The obtained results may help us to deeper understand the complex pathogenesis of the diseases (12). Because Alzheimer's disease neuroimaging initiative database contains multi-modality imaging data and genotyping data, most imaging genetic studies of brain disorders focus on Alzheimer's disease. Researchers applied the multi-task learning framework to discover several common brain regions of interests (ROIs) which are associated with the well-known AD-risk SNP (APOE rs429358) and disease status by multi-modality imaging fusion technology (13). Some methods utilized canonical correlation analysis to measure the association between multiple genetic variations and neuroimaging data (14). Though numerous studies focused on imaging genetics for AD and yielded some interesting results to understand the pathogenic mechanisms of AD, few studies focused on the imaging genetic association of MDD by using machine learning technology. Only some studies from clinicians usually use simple statistical analysis methods, such as ANVOVA analysis, correlation analysis, and mediation analysis, to analyze the association between the given SNP and brain regions from neuroimaging for MDD (15, 16). Despite these methods being simple, quick and easy, and useful, the machine learning methods in the recent medical imaging studies receives unprecedented breakthroughs (5) and have the ability to automatically and fully explore the association between genetic variations and neuroimaging.

In this study, our goal was to develop a simple yet powerful model for the automatic discovery of the association between genetic risk factors and disease status by using multi-modality neuroimaging data. Thus, we designed a novel imaging genetic association framework to mine the multi-modality phenotype network between genetic risk factors and multi-stage diagnosis status. This framework can not only identify consistent ROIs from multi-modality imaging data but also search new risk SNPs associated with MDD. In detail, the proposed approach consists of two steps: (i) Constructing the multi-modality phenotype network. The network of each subject consists of voxel node features and connectivity edge features, which are extracted from sMRI and rs-fMRI, respectively. (ii) Building the imaging genetic association model. Compared with existing simple MDD imaging genetic methods, our proposed association model utilized multi-task learning to explore the relationship between the MDD-risk SNP and the multi-modality phenotype network. Thereafter, multi-stage diagnosis status are embedded into the association model by a novel regularization term to fully use the internal relation across different modalities of different subjects. All the data were collected from the Affiliated Zhongda Hospital of Southeast University and the Second Affiliated Hospital of Xinxiang Medical University. The experimental results show that our method can not only improve the performance on metrics of root mean squared error and correlation coefficient but also identify a compact set of common ROIs across two brain network features, which are closed related to MDD genetic risk SNP TPH1 rs1799913. Moreover, some new and potential risk SNPs associated with MDD are discovered.

The contributions of this study are listed as follows:

1. An imaging genetic association model is proposed to fully explore the relationship between a given MDD-risk SNP TPH1 rs1799913 and the multi-modality phenotype network, which is constructed by voxel node features and connectivity edge features from sMRI and rs-fMRI, respectively.
2. The multistage diagnosis status is introduced into the associated model by a novel regularization term, which brings the ability to fully use the relationship across the multiple modalities of different subjects.
3. From our sequencing SNP genotype data, some new and potential risk SNPs associated with MDD is discovered, which can help researchers to further investigate the pathogenesis of MDD.

The rest of the article is organized as follows: Section 2 shows the demographic statistics of participants and pre-processing of multi-modality data. Section 3 presents our proposed method to mine the multi-modality phenotype network between genetic risk factors and multi-stage diagnosis status. Presentation and analysis of experimental results are shown in Section 4. Finally, discussion and conclusion are given in Section 5 and 6, respectively.

## 2. Data source and preprocessing

### 2.1. Participants

This study-utilized datasets were obtained from two hospitals, namely, the Affiliated Zhongda Hospital of Southeast University and the Second Affiliated Hospital of Xinxiang Medical University. The patients were recruited through the inpatient and outpatient departments of psychiatry in the mentioned two hospitals, while the healthy controls (HCs) were recruited from media advertising and community posting. The whole research procedures adhered to the Declaration of Helsinki. All patients signed the informed consent document and met an identical inclusion criteria as follows: (1) They met the criteria listed in Diagnostic and Statistical Manual of Mental Disorder (Fourth Edition); (2) they were in the first depressive episode, and the age of onset was over 18 years old; (3) they got a Hamilton Depression Scale-24 (HAM-D24) scores ≥20; (4) the absence of other major psychiatric illness history; (5) the absence of primary neurodegenerative disorders, including dementia or stroke; (6) no substance abuse or dependence (drug, caffeine, nicotine, alcohol, or others), head trauma, or loss of consciousness; (7) no cardiac or pulmonary disease which could influence the MRI scan. HC subjects met the (4) – (7) rules of the above inclusion criteria and were required to get a HAM-D24 score ≤ 8.

After removing poor quality images due to head motion or ghost intensity, this study contained 26 HCs and 45 patients with MDD from the Affiliated ZhongDa Hospital of Southeast University, and 38 HCs and 62 patients with MDD from the Second Affiliated Hospital of XinXiang Medical University. Thereafter, we utilized HAM-D24 scores to assess depression severity. A total of 107 patients with MDD (HAM-D24 scores ≥20) can be further divided into two subgroups: the HAM-D24 score of 20–34 points was defined as moderate depression (MD) and a HAM-D24 score of ≥35 was defined as severe depression (SD) (17). The detailed demographic of subjects are shown in Table 1.

**Table 1:** Demographic statics of subjects.

**Hospital**	**ZhongDa**			**XinXiang**		
**subjects**	**HC**	**MD**	**SD**	**HC**	**MD**	**SD**
Number	26	34	11	38	44	18
Gender(M/F)	10/16	15/19	4/7	21/17	26/18	7/11
Age	36.69 ± 13.88	11.09 ± 4.14	10.09 ± 5.99	10.74 ± 4.67	9.89 ± 4.25	9.44 ± 4.19
HAM-D24	1.27 ± 2.16	27.65 ± 4.68	38.91 ± 2.26	1.13 ± 1.85	29.91 ± 3.66	39.22 ± 3.95

HC, healthy control; MD, moderate depression; SD, severe depression; M/F, male/female; HAM-D24, Hamilton Depression Scale-24.

### 2.2. Magnetic resonance imaging (MRI) data acquisition and preprocessing

All participants underwent MRI scans at the baseline. MRI data were acquired using a 3.0 T Siemens scanner (Siemens, Erlangen, Germany) with a 12-channel head coil. The head of all subjects was immobilized with pads to minimize head movements. High-resolution 3D T1-weighted scan using a magnetization-prepared fast gradient echo (MPRAGE) sequence was performed according to the following parameters: repetition time (TR) = 1,900 ms, echo time (TE) = 2.48 ms, flip angle (FA) = 9°, acquisition matrix = 256 × 256, field of view (FOV) = 250 × 250 mm^2^, thickness = 1.0 mm, gap = 0, time = 4 min 18 s, and volume = 176. rs-fMRI parameters were as follows: TR = 2,000, msTE = 25 ms, FA = 90°, acquisition matrix = 64 × 64, FOV = 240 × 240 ^2^, thickness = 3.0 mm, gap = 0 mm, axial slice = 36, volume = 240; in-plane resolution parallel to the anterior-posterior conjunction = 3.75 × 3.75 ^2^, and acquisition time = 8 min. During the scan, subjects were asked to lie on their backs with their hands naturally resting on their sides. All subjects' heads were held in place with pads to minimize head movement. Ear plugs were used to reduce the noise of the scanner. Subjects were asked to relax their bodies, open their eyes, stay awake, and not think about anything specific to avoid falling asleep. Images were checked immediately after scanning to ensure quality, and scans were repeated if necessary.

For quality control, all image data were examined by two experienced radiologists. The rs-fMRI images were preprocessed using the Resting State Functional Data Processing Assistant (DPARSF 2.3 Advanced) MRI toolkit, which combines the Resting State Functional MRI Toolkit (REST, http://www.restfmri.net) and the Statistical Parametric Mapping Package (SPM, https://www.fil.ion.ucl.ac.uk/spm/) programs (18). The first 10 time points were excluded to ensure stable longitudinal magnetization and to accommodate the inherent scanner noise. The remaining 230 images were processed sequentially according to the following steps: (1) correction for time differences and head motion using the 36th slice as the slice time of the reference slice (participants with maximum head motion displacement greater than 1.5 mm in any direction [x, y, or z] or angular motion greater than 1.5° were excluded from the analysis); (2) T1 co-alignment with the functional image and subsequent reorientation; and (3) for spatial normalization, T1-weighted anatomical images were segmented into the white matter, the gray matter, and the cerebrospinal fluid and subsequently normalized to the Montreal Neurological Institute (MNI) space using transform parameters estimated by a uniform segmentation algorithm. These transform parameters were applied to the functional images, and the images were resampled with 3 mm isotropic voxels; (4) spatial smoothing was performed with a 4 mm full-width at half-peak (FWHM) isotropic Gaussian kernel; linear trends within each voxel time series were removed; interference signals (white matter, cerebrospinal fluid signals, and head motion parameters were calculated using rigid body six correction) and spiked regression volumes were regressed; and finally, a temporal bandpass (0.01–0.08 Hz) to minimize low-frequency drift and filter out high-frequency noise was performed.

### 2.3. SNP genotype data sequencing and processing

DNA genotyping was performed by Tianhao Biotechnology (Shanghai, China), and the standard protocol was employed to extract DNA from blood. The pre-designed Illumina next sequencing and array technology (Illumina Inc., San Diego, CA, USA) were utilized to determine the SNP genotypes in genes. Thereafter, we applied PLINK (v1.9) software to calculate the Hardy–Weinberg equilibrium (HWE) test, linkage disequilibrium statistics, and allele and genotype frequencies (19). After removing missing or incorrect values, the retained 5897 SNPs were used in this study.

Genetic risk variants can help researchers understand the relevant diseases of biological mechanism and provide an effective hypothesis for drug design. In this study, we focused on fully to explore the relationship between a given risk SNP and quantitative traits of the brain structure and functional level. Some researchers have implicated a large number of gene-related depression, including CACNA1E, BDNF, CRHR1, GSK3β, TPH1, and so on, see those in the systematic review (20). However, different from Alzheimer's disease with a well-known risk SNP APOE rs429358, there were no consistent gene hypotheses for the pathogenesis of MDD. SNPedia (http://www.SNPedia.com) is a wiki resource of the functional consequences of human genetic variation as published in peer-reviewed studies. We searched the related genetic-risk SNP associated with MDD in the SNPedia database. Among the retained 5897 SNPs, only TPH1 rs1799913 is successfully matched. Tryptophan hydroxylase (TPH) is the rate-limiting enzyme in the biosynthesis of serotonin (5-HT) and haplotype analysis indicates that TPH-1 associates with MD. In literature (21), six SNPs were found at linkage disequilibrium in both patients and control subjects, but only one SNP (rs1799913) significantly associated with MD by single marker association analysis. This SNP, also known in the literature as SNP A779C, has been associated with cerebrospinal fluid (CSF) 5-hydroxyindoleacetic acid (5-HIAA) concentrations, and several reports have shown its association with suicidal behavior (22, 23).

Thus, TPH1 rs1799913 was used in the following experiments to verify our proposed model. TPH1 rs1799913 value was coded in an additive fashion as 0, 1, and 2, where the alleles were divided into major and minor alleles by genotype frequency, and the major allele was coded as “0,” the minor allele as “2,” and the heterozygote of the remaining alleles as “1” (19).

## 3. Method

**Figure 1:**
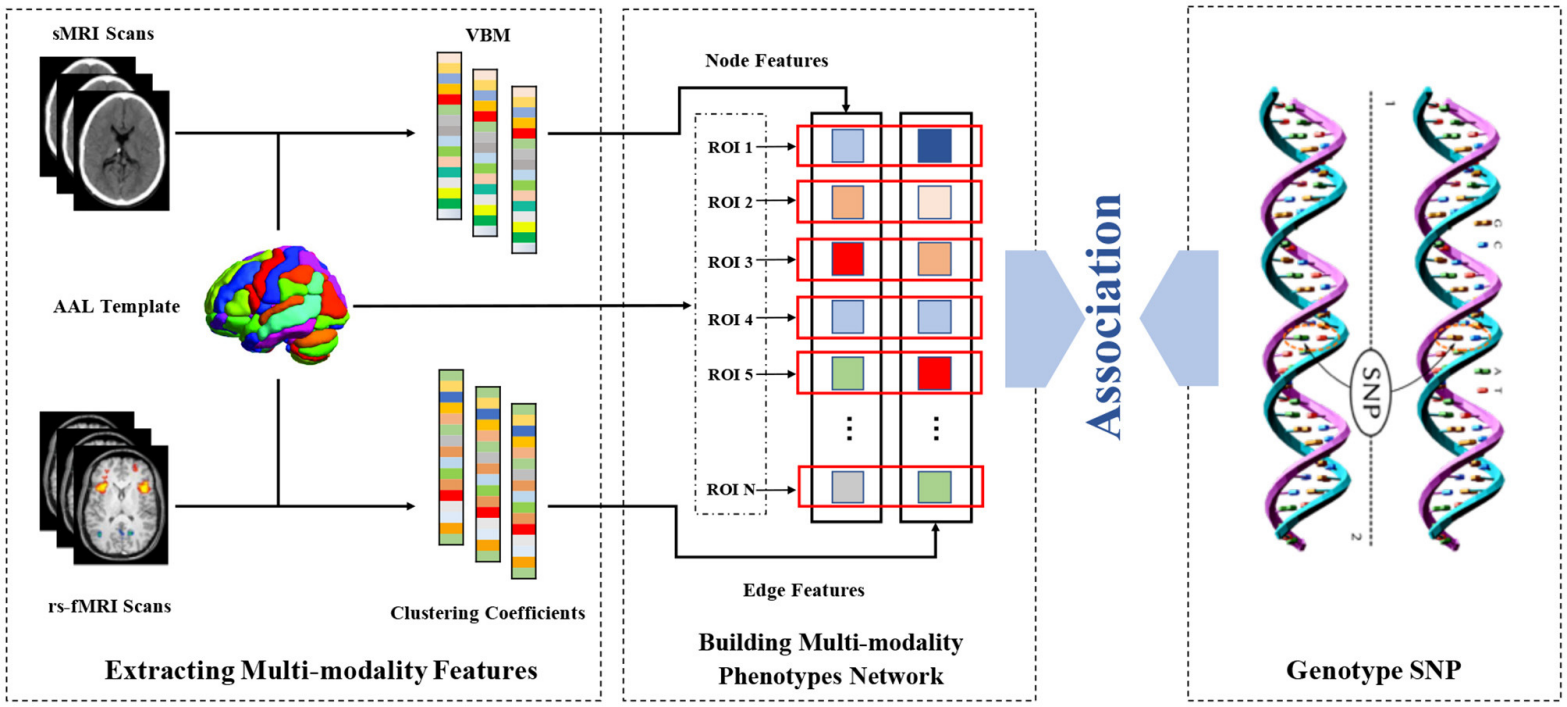
The overview of our proposed model.

Recently, most of the neuroimaging genomic studies using machine learning technology focus on Alzheimer's disease, and few studies focus the pathogenesis of MDD. These methods usually research the association between genetic variants and structural imaging but ignore the functional connectivity information between various brain regions. As it is well-known, the human brain is a highly complex network, which contains higher-order connectivity information to help understanding the pathogenesis of diseases. Therefore, our model utilized the brain rs-fMRI and sMRI of each subjects as the input. The overview of our proposed model is shown as Figure 1. First, the network voxel node features and connectivity edge features were extracted from sMRI and rs-fMRI scans based on Automated Anatomical Labeling (AAL), respectively. After that, a multi-modality association model-introduced multi-stage diagnosis status of subjects was proposed to fully explore the relationship between two brain features and a given risk MDD SNP (TPH1 rs1799913) in high confidence.

### 3.1. Building the multi-modality phenotype network

In this study, each subject builds a multi-modality brain phenotype network. The node and edge features of brain network were extracted from sMRI and rs-fMRI, respectively.

After the preprocessing sMRI data, the voxel-based morphometry (VBM) of each subject were obtained from the normalized gray matter density maps, which were created in the MNI space as 2 × 2 × 2*mm*^3^ voxels. Thereafter, we aligned VBM to each participant's same visit scan and further extracted 116 ROI level measurements of mean gray matter densities based on the AAL template. In our proposed method, each ROI is modeled as a single node of multi-modality brain network. Thus, each subject could produce one set of node features.

For rs-fMRI data, a functional connectivity network usually is constructed for representing each subject, with each node denoting a pre-defined brain ROI and each edge representing the pairwise functional connection between ROIs. In this study, we extracted the mean time series of each ROI based on the AAL template and normalized them with zero mean and unit variance. After that, functional connectivity networks were generated by using the Pearson correlation coefficient and hence could capture the correlation between the BOLD signals of paired ROIs.

Due to the psychiatric disorders with abnormal topological properties of brain networks, many graph theory-based methods have played an important role in the human brain disorder analysis. Among them, clustering coefficient (CC) is one of the most popular methods and reflects local clustering properties of the brain network (24). Therefore, after constructing the functional connectivity network, the clustering coefficients can be calculated by


(1) $$\begin{array}{l} CC^{W}\left(i\right)=\frac{2}{k_{i}\left(k_{i}-1\right)}\underset{j,h}{\sum }\left(\bar{w_{ij}}\cdot \bar{w_{ih}}\cdot \bar{w_{jh}}\right), \end{array}$$

where *w*_*ij*_, *w*_*ih*_, and *w*_*jh*_ are the connection weights between node *i* and *j*, between node *i* and *h*, and between node *j* and *h*, respectively. Scaled for weights $\bar{w_{ij}}$, $\bar{w_{ih}}$, and $\bar{w_{jh}}$, that is, $\bar{w_{ij}}\leftarrow w_{ij}/max\left(w\right)$, $\bar{w_{ih}}\leftarrow w_{ih}/max\left(w\right)$, $\bar{w_{jh}}\leftarrow w_{jh}/max\left(w\right)$, *max*(*w*) denotes the maximum connection weight in the brain network. The number of edges connected to node *i* is denoted by *k*_*i*_. Finally, we extracted a set of clustering coefficients from the functional connectivity networks as the connectivity edge furthers of the multi-modality phenotype network. Thus, each subject could produce one set of edge features.

### 3.2. Associations between genotype and multi-modality phenotype network

After building the multi-modality phenotype network of each subject, pathological alterations are considered to be abnormal changes in phenotype networks. Since the network node and edge features consist of VBM from sMRI and clustering coefficients from rs-fMRI, abnormal changes are closely related to associated ROIs and significant connectivity edges. In this study, we suppose that there are N subjects, with each one represented by a multi-modality phenotype network. Given *M* modalities of phenotypes $X^{m}={\left[X_{1}^{m},...,X_{n}^{m},...,X_{N}^{m}\right]}^{T}\in R^{N\times d}$ as the input and the corresponding response value() $y={\left[y_{1},...,y_{n},...,y_{N}\right]}^{T}\in R^{N}$ as the output, where *d* is the number of node and edge features dimensionality. Let *w*^*m*^∈*R*^*d*^ denote the linear discriminant function corresponding to the *m*-th modality. Then the multi-modality network phenotype association model can be formulated as:


(2) $$\begin{array}{l} \underset{W}{min}\frac{1}{2}\overset{M}{\underset{m=1}{\sum }}||y-X^{m}w^{m}|{|}_{2}^{2}+\lambda ||W|{|}_{2,1}, \end{array}$$

where *W* = [*w*^1^, *w*^2^, ..., *w*^*M*^]∈*R*^*d*×*M*^ is the weight matrix, and each row *w*_*j*_ represents the vector of coefficients assigned to the *j*-th features across multiple modalities. It is worth noting that Equation (2) adds the *L*_2, 1_-norm regularization term, $||W|{|}_{2,1}=\overset{d}{\underset{j=1}{\sum }}||w_{j}|{|}_{2}$, which is a “group-sparsity” regularizer and penalizes all coefficients in the same row of matrix *W* for joint feature selection. This means that our proposed modal can force only a small number of features to be selected across multiple modalities. The regularization parameter λ is used to balance the contributions of two terms in Equation (2). The larger the value of λ, the fewer the features are selected.

### 3.3. Introduction multi-stage diagnosis status into the association model

One disadvantage of the aforementioned multi-modality association model is that it only considers the relationship between the multiple modalities of the same subject and ignores the relationship between imaging phenotypes and diagnosis status among subjects. In order to overcome this limitation, we made full use of the multi-stage diagnosis status of each subjects, i.e., HC, SD, and MD, and then introduced a novel regularization term that can embed the multi-stage diagnosis status of MDD:


(3) $$\begin{array}{l} \overset{N}{\underset{i,j}{\sum }}||{\left(w^{m}\right)}^{T}x_{i}^{m}-{\left(w^{m}\right)}^{T}x_{j}^{m}|{|}_{2}^{2}S_{ij}^{m}=2{\left(w^{m}\right)}^{T}{\left(X^{m}\right)}^{T}L^{m}X^{m}w^{m}, \end{array}$$

where *L*^*m*^ = *D*^*m*^−*S*^*m*^ denotes a Laplacian matrix for the *m*-th modality, where *D*^*m*^ is the diagonal matrix and each element is defined by $D_{ij}^{m}=\overset{N}{\underset{j=1}{\sum }}S_{ij}^{m}$. In this study, $S=\left[S_{ij}^{m}\right]\in R^{n\times n\times m}$ represents a similarity matrix which measures the similarity between each pair of subjects on the *m*-th modality and can be defined as


(4) $$S_{ij}^{m}=\{\begin{array}{ll} 1, & {\text{if x}}_{\text{i}}^{\text{m}}{\text{ and x}}_{\text{j}}^{\text{m}}\text{are from the same class in m-th modality}\\ 0, & \text{otherwise}. \end{array}$$

The similarity between subjects within the same class and modality can be defined as 1; otherwise, it is 0. The purpose of Equation (3) is to enforce these subjects from the same class and modality to be close to each other in the label space. When $x_{i}^{m}$ and $x_{j}^{m}$ are from the same class in the *m*-th modality, the distance between ${\left(w^{m}\right)}^{T}x_{i}^{m}$ and ${\left(w^{m}\right)}^{T}x_{j}^{m}$ should be as small as possible in the label space.

In this study, we introduced the multi-stage diagnosis status into the multimodality association model by incorporating the regularizer (3) into Equation (2). The objective function of our proposed association model (MSD-MM) can be formulated as follows:


(5) $$\begin{array}{l} \underset{W}{min}\frac{1}{2}\sum_{m=1}^{M}{\left\|y-X^{m}w^{m}\right\|}_{2}^{2}+\lambda_{1}{\left\|W\right\|}_{2,1}+\lambda_{2}\sum_{m=1}^{M}{\left(w^{m}\right)}^{T}\\ \begin{array}{l} {\left(X^{m}\right)}^{T}L^{m}X^{m}w^{m}, \end{array} \end{array}$$

where parameter λ_1_ and λ_2_ are used to control two regularization terms, respectively. Their values can be determined by inner cross-validation on training data. From the objective function Equation (6), the MSD-MM model can not only jointly select a sparse subset of common features from multi-modality data but also fully use the prior diagnosis information among subjects. To efficiently solve the objective function in Equation (6), we used the Nesterov's accelerated proximal gradient optimization algorithm (25).

## 4. Experimental results and analysis

### 4.1. Experimental settings

In our experiments, we adopted two evaluation metrics, i.e., root mean squared error (RMSE) and correlation coefficient (CC), which are wildly used to measure performance regression and association analysis between the predicted and actual response values, respectively.

The five-fold cross validation strategy is implemented to validate the effectiveness of our proposed method. For the parameters λ_1_ and λ_2_ of regularization in Equation (5), we tuned them from 10^−5^, 3 × 10^−5^, 10^−4^, 3 × 10^−4^, ..., 3 and determined their values by the nested five-fold cross validation on the training dataset.

In this study, we compared the single-modality (SM) method, concatenate-modality (CM) method, and multimodality (MM) method with/without multi-stage diagnosis status. In Table 2, SM, CM, and MM are conventional methods without diagnosis status. Since one contribution of this study is the introduction of diagnosis status, which provide more prior knowledge, MSD-SM and MSD-CM are improved SM and CM methods incorporating multi-stage diagnosis status, respectively. MSD-MM is our proposed method, which simultaneously considers the multi-modality images and multi-stage diagnosis status. The detailed description of various comparisons are shown in Table 2.

**Table 2:** Detailed descriptions of various comparisons.

**Comparison**	**Modality**	**Diagnosis**	**Description**
SM	Node	No	Using Lasso (Least absolute shrinkage and selection operator) to detect a sparse significant subset from node or edge features.
SM	Edge	No	
MSD-SM	Node	Yes	
MSD-SM	Edge	Yes	
CM	-	No	Concatenating node and edge features, and then using Lasso to detect a sparse significant subset from combined features.
MSD-CM	-	Yes	
MM	Node	No	Detecting a sparse subset of common ROIs from node and edge features.
MM	Edge	No	
MSD-MM	Node	Yes	
MSD-MM	Edge	Yes	

### 4.2. Association between risk SNP and the multi-modality network phenotype

We compared our proposed MSD-MM method with conventional methods without diagnosis status (including SM, CM, and MM) and improved methods with diagnosis status (including MSD-SM and MSD-CM). In order to eliminate the bias of random division, we performed five times independent and non-repetitive five-fold cross validation. Thereafter, the average results of RSEM and CC on the training and testing data on node and edge modalities were calculated, respectively, as shown in Table 3.

**Table 3:** Comparison of regression performance on risk SNP TPH1 rs1799913 by different methods.

**Method**		**RMSE (mean** ±**std)**		**CC (mean** ±**std)**	
		**Train**	**Test**	**Train**	**Test**
SM	Node	1.3107 ± 0.1007	1.3644 ± 0.1654	0.0465 ± 0.0440	0.0121 ± 0.0045
SM	Edge	1.3454 ± 0.2189	1.5456 ± 0.1566	0.0983 ± 0.0564	0.0488 ± 0.0027
MSD-SM	Node	0.7030 ± 0.0564	1.0114 ± 0.1291	0.4340 ± 0.0617	0.2164 ± 0.0945
MSD-SM	Edge	1.1610 ± 0.0790	1.3726 ± 0.2136	0.3029 ± 0.0594	0.2196 ± 0.1448
CM	-	1.3988 ± 0.0949	1.4245 ± 0.1314	0.0984 ± 0.0654	0.0745 ± 0.0915
MSD-CM	-	0.8736 ± 0.0654	1.3034 ± 0.1195	0.4021 ± 0.0390	0.2254 ± 0.0966
MM	Node	0.6144 ± 0.1647	1.1348 ± 0.1649	0.5416 ± 0.0412	0.1654 ± 0.0925
MM	Edge	0.8117 ± 0.1054	1.2654 ± 0.1267	0.4645 ± 0.0561	0.1714 ± 0.0729
MSD-MM	Node	0.6887 ± 0.0653	0.9685 ± 0.1308	0.4971 ± 0.0712	**0.2432 ± 0.0799**
MSD-MM	Edge	0.8310 ± 0.1410	1.1892 ± 0.2271	0.3625 ± 0.0254	**0.2697 ± 0.0910**

This means that two results of the bold vales are significantly better than the above results.

As shown in Table 3, MSD-SM receives the RMSE values of 1.0114 and 1.3726 and the CC values of 0.2164 and 0.2196 on the node and edge features, respectively, achieving better performance than the conventional SM method. MSD-CM obtains the RMSE value of 1.3034 and the CC value of 0.2254, which are better than those of the CM method. MSD-MM yields the best RMSE values of 0.9685 and 1.1892 and the CC values of 0.2432 and 0.2697 on the two different features. These results indicate three findings: 1) Compared with the SM-type method, the MM-type method can jointly select node and edge features and significantly improve the performance of regression and association analysis; 2) after introducing multistage diagnosis information, the proposed MSD-type method consistently outperform their conventional methods in both RMSE and CC performance measurements; 3) compared with voxel-based morphometry node features, the functional connectivity edge features between different brain regions provide more insights for the mechanistic understanding of MDD; moreover, CM-type and MM-type methods both utilize node and edge features, but they apply distinct strategies to combine two features and receive different performance. The CM-type method directly concatenate node and edge features. This way may be lost when the relationship information of two modalities and bring more noise in widespread feature space, while the MM-type method uses the multi-task strategy (*L*_2, 1_-norm constrain) to jointly select node and edge features, which can improve the robustness of ROIs detection. In a word, our proposed MSD-MM method yields the best performance on RMSE and CC measures. That demonstrates that simultaneously considering multi-modality imaging data and multi-stage diagnosis status can improve the performances of regression and association analysis between the imaging phenotype and genotype.

### 4.3. Identification of the related node ROI markers from sMRI data

In addition to improving the measure performances of regression and association analysis, one major purpose of this study is to identify some significant imaging phenotypes, which are highly associated with both risk SNP and multi-stage diagnosis status. In this study, due to the use of multi-modality imaging data, these identified phenotypes offer the possibility of detecting associations between the genotype and the brain structure as well as the function and help researchers further understand the pathogenesis of MDD.

**Figure 2:**
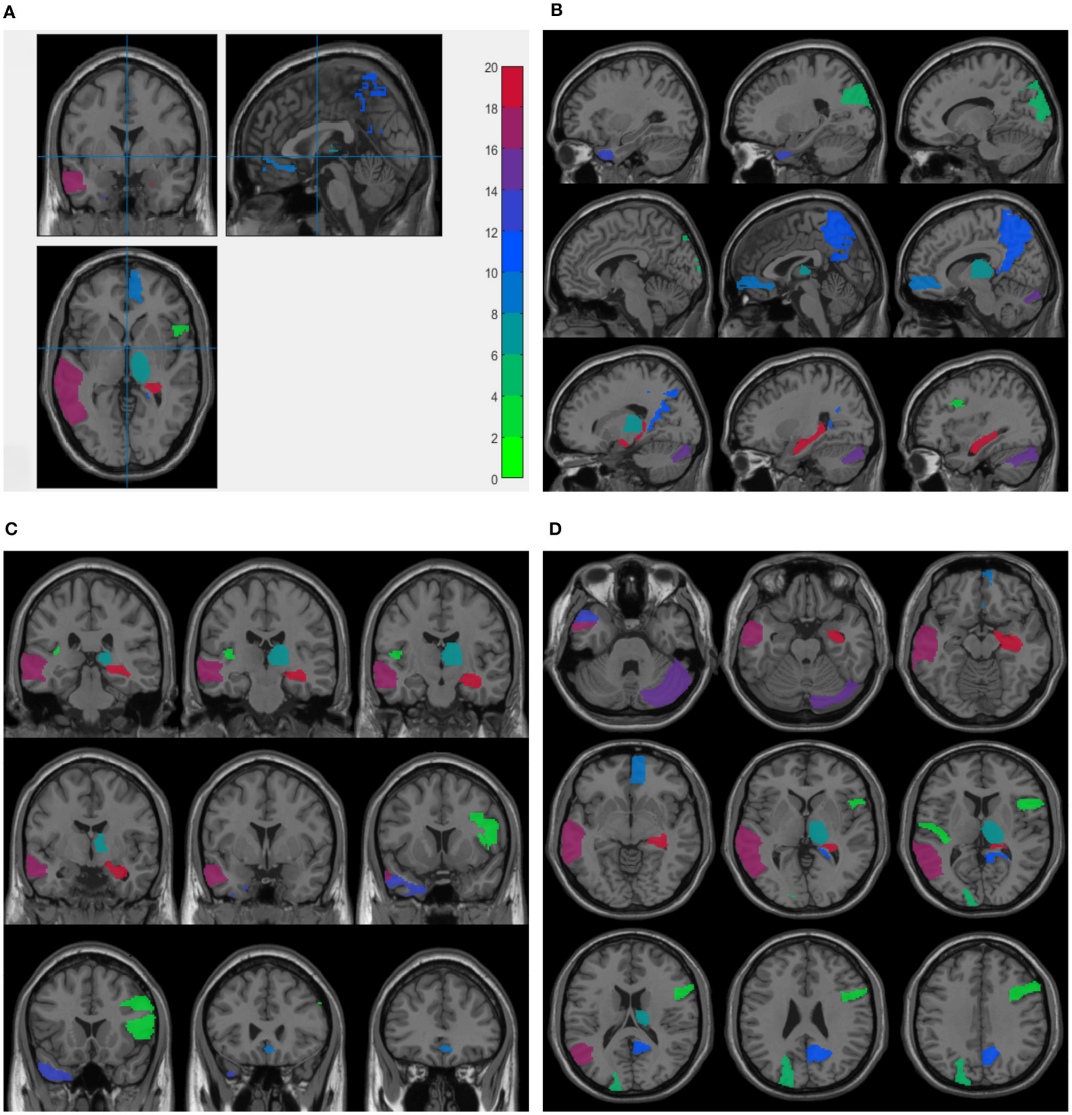
Visualization of the top 10 ROIs selected by node features in three planes: Three planes of sMRI scan **(A)**, sagittal plane **(B)**, coronal plane **(C)**, and axial plane **(D)**. The colors represents the average weight values of the corresponding ROI markers. These figures are plotted.

For node features from sMRI data, we averaged the weight values by five times repeated five-fold cross validations and selected the top 10 maximum weight ROIs as the significant ROI markers. Table 4 presents the top 10 selected ROIs from sMRI data and the corresponding average weight values. Next, these weight values were mapped onto the human brain, and Figure 2 shows the visualization of the top 10 selected ROIs in all three planes (sagittal, coronal, and axial). In each plane, the colors of the labeled brain regions reflect the average weight values of the corresponding selected ROIs. The remarkable thing is that most of the selected ROIs are consistent with earlier findings, which focus on structural images and have already identified several diagnostic markers of MDD. The literature (26) indicates that patients with MDD exhibit bilateral volume reduction in major hippocampal substructures and identify core hippocampal regions in MDD pathology as a potential marker of disease progression in MDD. Moreover, researchers find that reduced hippocampal gray matter volume is a common feature of patients with MDD (27). The bilateral middle frontal gyrus shows that the amplitude of low-frequency fluctuation (ALFF) significantly increases in subjects with subclinical depression (16, 28). Structural abnormalities in the thalamus might be the potential trait marker of MDD at the early stage as in (29, 30). Compared with healthy controls, patients with MDD presents decreased gray matter density in the bilateral temporal pole and right superior temporal gyrus (31).

**Table 4:** Top 10 ROIs selected by the node features from sMRI data.

**ID**	**ROI name**	**Weight**
38	Hippocampus.R	6.78
85	Temporal Mid.L	3.73
92	Cerebelum Crus1.R	3.32
87	Temporal Pole Mid.L	3.05
68	Precuneus.R	2.98
10	Frontal Mid Orb.R	2.78
78	Thalamus.R	2.64
49	Occipital Sup.L	2.03
79	Heschl.L	1.90
12	Frontal Inf Oper.R	1.70

### 4.4. Identification of the related edge ROIs marker from rs-fMRI data

The brain system can be simply represented by a brain network model, whose nodes and edges are defined as brain regions and connections between brain regions, respectively. In this study, rs-fMRI data are used to construct the brain functional connectivity network. After that, the clustering coefficients are extracted as edge features and are absorbed into each brain region. Thus, the dimension of the obtained edge features is the same as the number of the brain regions, and each dimension corresponds to one brain region. Thus, we can identify the related ROI markers from rs-fMRI data by our proposed method.

For edge features from rs-fMRI data, we also averaged the weight values by five times repeated five-fold cross validations and selected the top 10 maximum weight ROIs as the significant ROI markers, as shown in Table 5. Some existing studies using rs-fMRI data have identified several diagnostic brain region markers for MDD, such as precuneus, cingulum and temporal, which are also consistent with the aforementioned selected relevant ROIs. The study (32) shows that guilt-selective to MDD is associated with functional disconnection of anterior temporal and subgenual cortices. Patients with MDD have an abnormal activity of the precuneus at the resting state in first-episode drug-naive. This indicates that activity within the precuneus may be a potential biomarker for the diagnosis of MDD (33). In literature (34), the increased fractional amplitude of low-frequency fluctuation (fALFF) in the left mid cingulum, right precuneus, and left superior frontal gyrus may serve as a neuroimaging marker for first-episode MDD. The combined use of the increased fALFF in the right precuneus and left superior frontal gyrus obtains the best diagnostic scores. Compared to patients with remitted MDD and to healthy controls, patients with recurrent MDD exhibit decreased fALFF in the right posterior insula and right precuneus and increased fALFF in the left ventral anterior cingulate cortex (35).

**Table 5:** Top 10 ROIs selected by the edge features from rs-fMRI data.

**ID**	**ROI name**	**Weight**
85	Temporal Mid.L	8.47
32	Cingulum Ant.R	8.05
89	Temporal Inf.L	6.78
68	Precuneus.R	6.52
54	Occipital Inf.R	5.59
38	Hippocampus.R	2.46
33	Cingulum Mid.L	1.95
35	Cingulum Post.L	1.69
41	Amygdala.L	1.60
30	Insula.R	1.59

**Figure 3:**
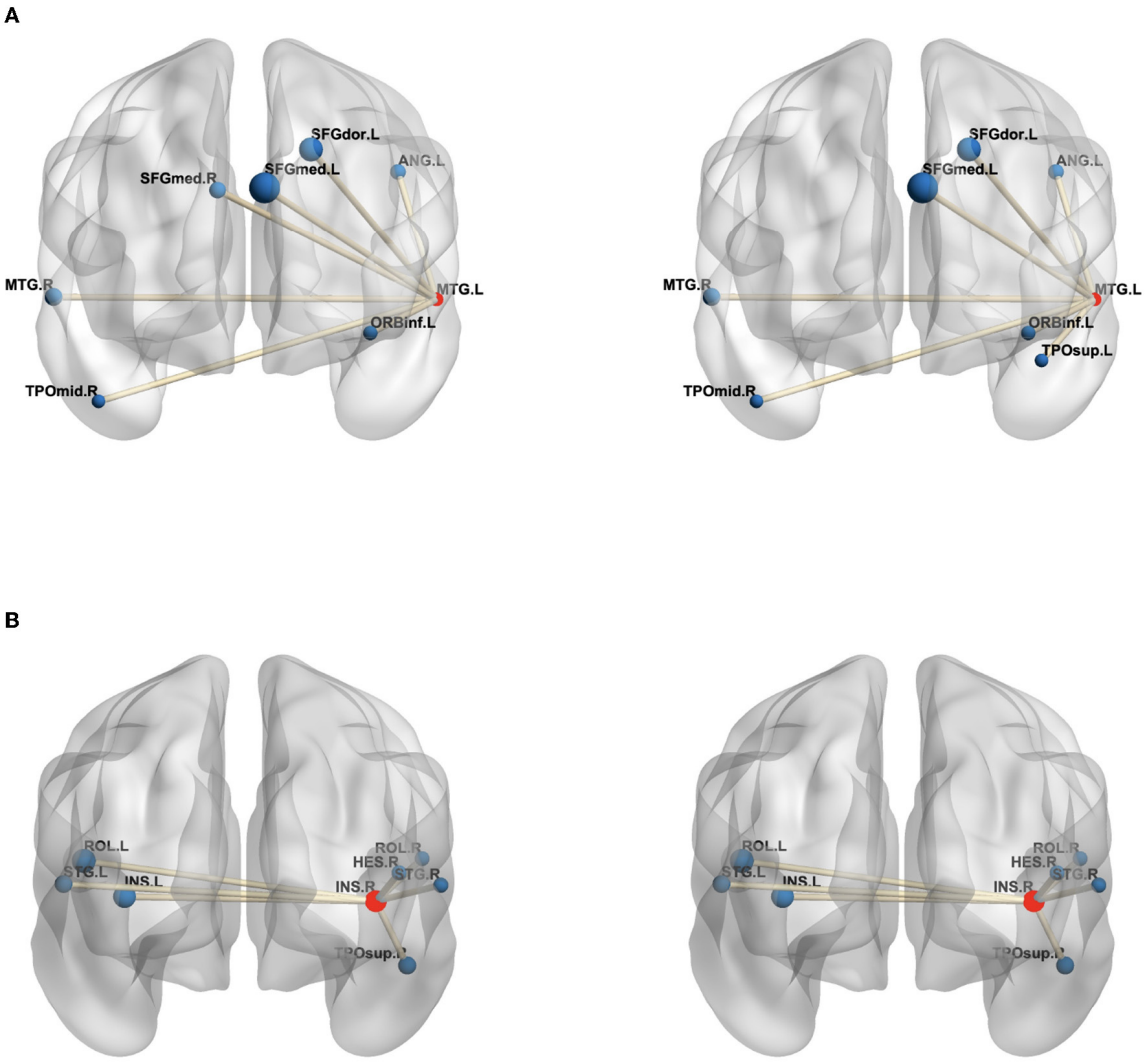
The edges of the maximum weight ROI **(A)** and minimum weight ROI **(B)** on the MDD (left) and HC (right) group. The centroid red node represents the selected ROI, and the blue node denotes the corresponding ROI linked by top seven average connection value edges. **(A)** MTG.L, Temporal Mid gyrus.L; MTG.R, Temporal Mid.R; ANG.L, Angular.L; SFGdor.L, Frontal Sup.L; SFGmed.L, Frontal Sup Medial.L; SFGmed.R, Frontal Sup Medial.R; ORBinf.L, Frontal Inf Orb.L; TPOmid.R, Temporal Pole Mid.R; TPOsup.L, Temporal Pole Sup.L; **(B)** INS.R, Insula.R; INS.L, Insula.L; HES.R, Heschl.R; ROL.L, Rolandic Oper.L; ROL.R, Rolandic Oper.R; STG.L, Temporal Sup.L; STG.R, Temporal Sup.R; TPOsup.R, Temporal Pole Sup.R. In the figure, all edges of each brain figure are plotted by BrainNet (36).

Moreover, in order to analyze the functional connectivity of selected brain regions and graphically compare the difference on the functional connectivity network between patients with MDD and HCs, we selected the maximum weight ROI (left temporal) and the minimum weight ROI (right Insula) in Table 4 and then calculated the average edge values of functional connectivity network for MDD and HC group, respectively. Specifically, we first constructed the functional connectivity networks of each subject in MDD and HC group. After that, the average functional connectivity networks were calculated for each group. Finally, we selected seven edges with the highest connection values from all edges of a given brain region (14). Figure 3 graphically presents the top seven average connection value edges on maximum and minimum weight ROIs. As seen in Figure 3, compared to the HC group, the edges of maximum weight ROI in the MDD group have a distinct change, which is that the edge connecting right temporal middle and right frontal superior medial is replaced to the edge connecting right frontal superior medial and left temporal pole superior. However, the edges of the minimum weight ROI in the MDD group are same as those in the HC group. The aforementioned results it demonstrate that the identified significant brain regions (the maximum weight ROI) are highly correlated to the pathogenesis of MDD.

### 4.5. Identification of the consistent ROIs from multi-modality imaging data

**Figure 4:**
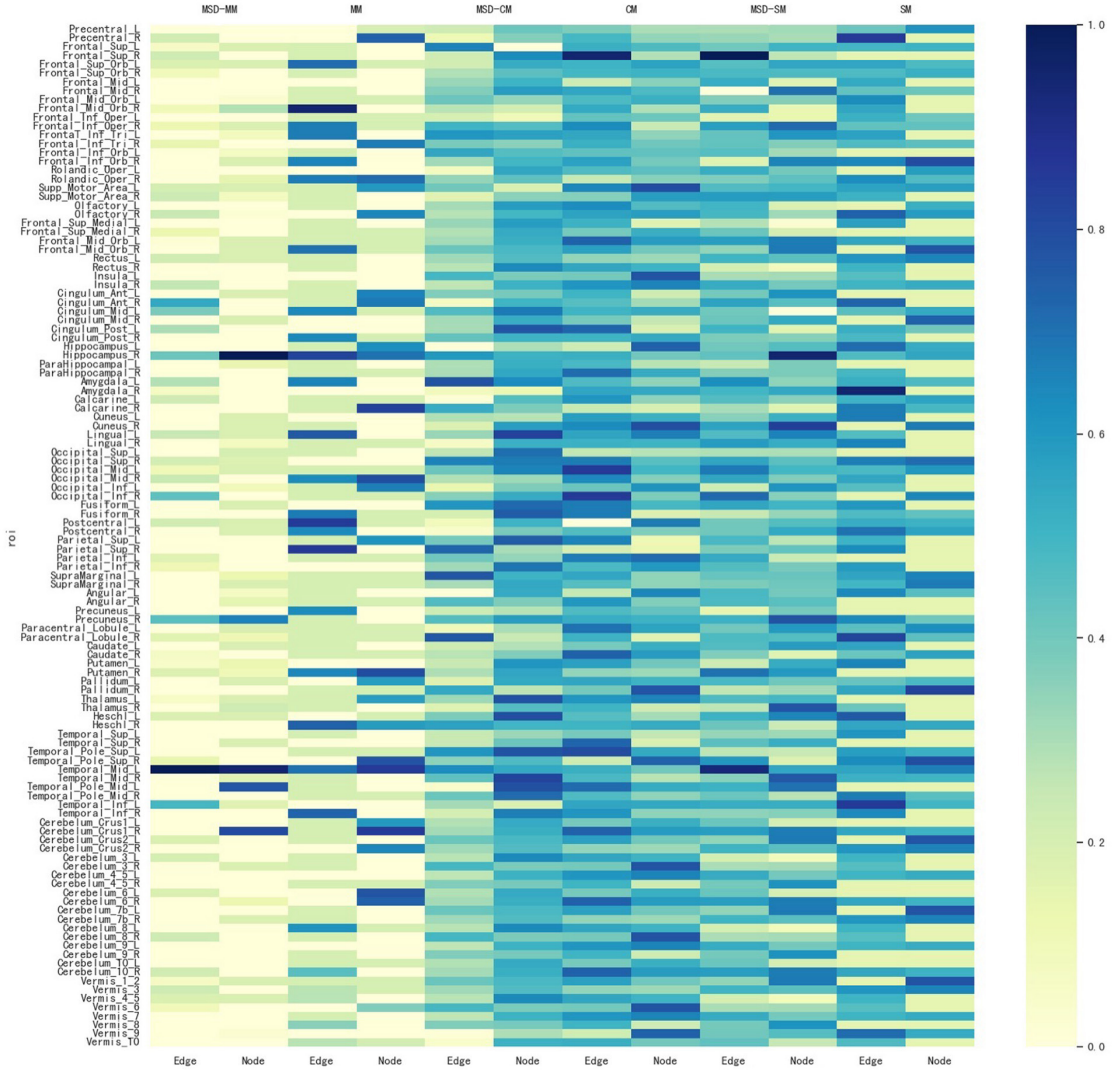
Weight maps for the multi-modality imaging data on 116 ROI associated with SNP THP1 rs1799913 respect to different methods.

In addition, for identifying some significant ROIs from node and edge features, another advantage of our proposed method is to ensure that these identified brain regions are consistent in both node and edge features. Figure 4 shows all comparisons of weight maps for multi-modality data on 116 ROIs associated with risk SNP TPH1 rs11179027. As seen in Figure 4, SM-based and CM-based methods select a large number of ROIs, while these ROIs are inconsistent across node and edge features. This means that researchers find it hard to use the selected ROIs for further investigation. However, the MSD-MM method is able to identify sparse and consistent ROIs associated with THP1 rs11179027 using multi-modality imaging data. These identified ROIs, such as left temporal, right hippocampus, and right precuneus parts, strongly agree with the existing studies and are highly correlated with MDD (26, 27, 33, 37, 38). To sum up, our proposed method tends to select consistent ROIs associated with risk SNP across multi-modality imaging data, which show a great value to further investigate the mechanism of MDD.

### 4.6. Identification of new risk SNPs associated with MDD

As is well-known, the pathogenesis of MDD may be caused by a large number of genetic risk SNPs. However, we only explored the relationship between two brain network features and a given MDD-risk SNP, THP1 rs1799913 in the aforementioned experiments and then identified some significant brain ROIs associated with this SNP. Results demonstrate that our proposed method can be used as an effective tool to mine new risk SNPs associated with MDD. In this study, the genotype data contains 5879 SNPs. In this study, we performed the MSD-MM method for each SNP in the whole genotype data. Table 6 shows that four SNPs have similar correlation coefficient with THP1 rs1799913 on node and edge features, respectively. This means that four SNPs may be risk genetic SNP associated with MDD. The literature (39) presents that PIK3R1 rs3730089 is related to schizophrenia and bipolar disorder in the Han Chinese population, but patients with schizophrenia, bipolar disorder, and MDD usually show common symptoms, such as anhedonia and amotivation. Recent research studies systematically report that these three mental disorders have a familial clustering character and any two or even three of these disorders could co-exist in some families. In addition, evidence from symptomatology and psychopharmacology also imply that there are intrinsic connections between these three mental disorders (40). Thus, PIK3R1 rs3730089 may be a risk SNP marker in MDD research. Meanwhile, although KDSR rs1138488, LAMA2 rs2229848, and THY1 rs3138094 receive fine correlation coefficients by the MSD-MM method, now there are no medical or biological research studies that directly support three SNPs related to MDD, and we expect that to be verified in future studies. We hope that these new MDD-risk SNPs will be verified in future studies and give more insights to understand the pathogenesis of MDD.

**Table 6:** Correlation coefficient of four new SNPs on node and edge features by the MSD-MM method.

**SNPs**	**Edge**	**Node**
rs3730089 (PIK3R1)	0.2033 ± 0.1300	0.2118 ± 0.0355
rs1138488 (KDSR)	0.2032 ± 0.0945	0.2209 ± 0.0245
rs2229848 (LAMA2)	0.2143 ± 0.1003	0.2170 ± 0.1370
rs3138094 (THY1)	0.2248 ± 0.1233	0.2235 ± 0.1281

## 5. Discussion

### 5.1. Risk MDD SNP vs. non-disease related SNP

In the SNPedia database, TPH1 rs1799913 has been confirmed as a genetic risk variant associated with MDD. We evaluated the performance of regression and association analysis on the risk SNP TPH1 rs1799913 by the MSD-MM method in the aforementioned experiments, as shown in Table 3. To verify that the improvement is brought by only using the risk MDD SNP, we further selected three non-MDD related SNP for comparison. TPH2 rs11179027 is the nearest SNP to TPH1 rs1799913 (41), APOE rs429358 is a well-known top risk SNP associated with AD (42), and AKT3 rs14403 is a genetic risk SNP for schizophrenia (43). The MSD-MM method evaluated correlation coefficients of three non-MDD related SNPs, and results of all comparisons on the test dataset are shown in Table 7. Compared to the risk SNP THP1 rs1799913 reported in Table 3, all comparisons (including MSD-MM method) receive very low correlation coefficient values. The reason of the poor performance is that the train data containing the non-related MDD SNP lead to an overfitting problem for all comparisons and then lose the power of generalization on the test data. Thus, the contrast experiment shows that the learned consistent multi-modality imaging phenotypes if and only if using the risk MDD SNP can discovery the potential biological pathway from gene to the brain for clinical diagnosis.

**Table 7:** Correlation coefficients of all comparisons for three non-MDD related SNPs on the test data.

**Method**		**rs11179027**	**rs429358**	**rs14403**
SM	Node	0.0102 ± 0.0951	−0.0239 ± 0.0236	0.0933 ± 0.0320
SM	Edge	−0.0323 ± 0.1005	0.0482 ± 0.0319	0.0209 ± 0.0334
MSD-SM	Node	0.0290 ± 0.1118	−0.0254 ± 0.0282	0.0089 ± 0.0101
MSD-SM	Edge	−0.0199 ± 0.1366	0.0358 ± 0.0234	0.0357 ± 0.0176
CM	-	0.0156 ± 0.0991	0.0431 ± 0.0341	0.0298 ± 0.0425
MSD-CM	-	0.0095 ± 0.1668	0.0525 ± 0.1934	0.0290 ± 0.2443
MM	Node	0.0346 ± 0.0160	-0.0565 ± 0.0349	0.0233 ± 0.0218
MM	Edge	−0.0211 ± 0.1519	0.0323 ± 0.1843	0.0179 ± 0.0024
MSD-MM	Node	0.0344 ± 0.1274	−0.0887 ± 0.0034	0.0445 ± 0.0367
MSD-MM	Edge	−0.0104 ± 0.1351	0.0522 ± 0.0337	0.0603 ± 0.0285

### 5.2. The selection of regularization parameters

**Figure 5:**
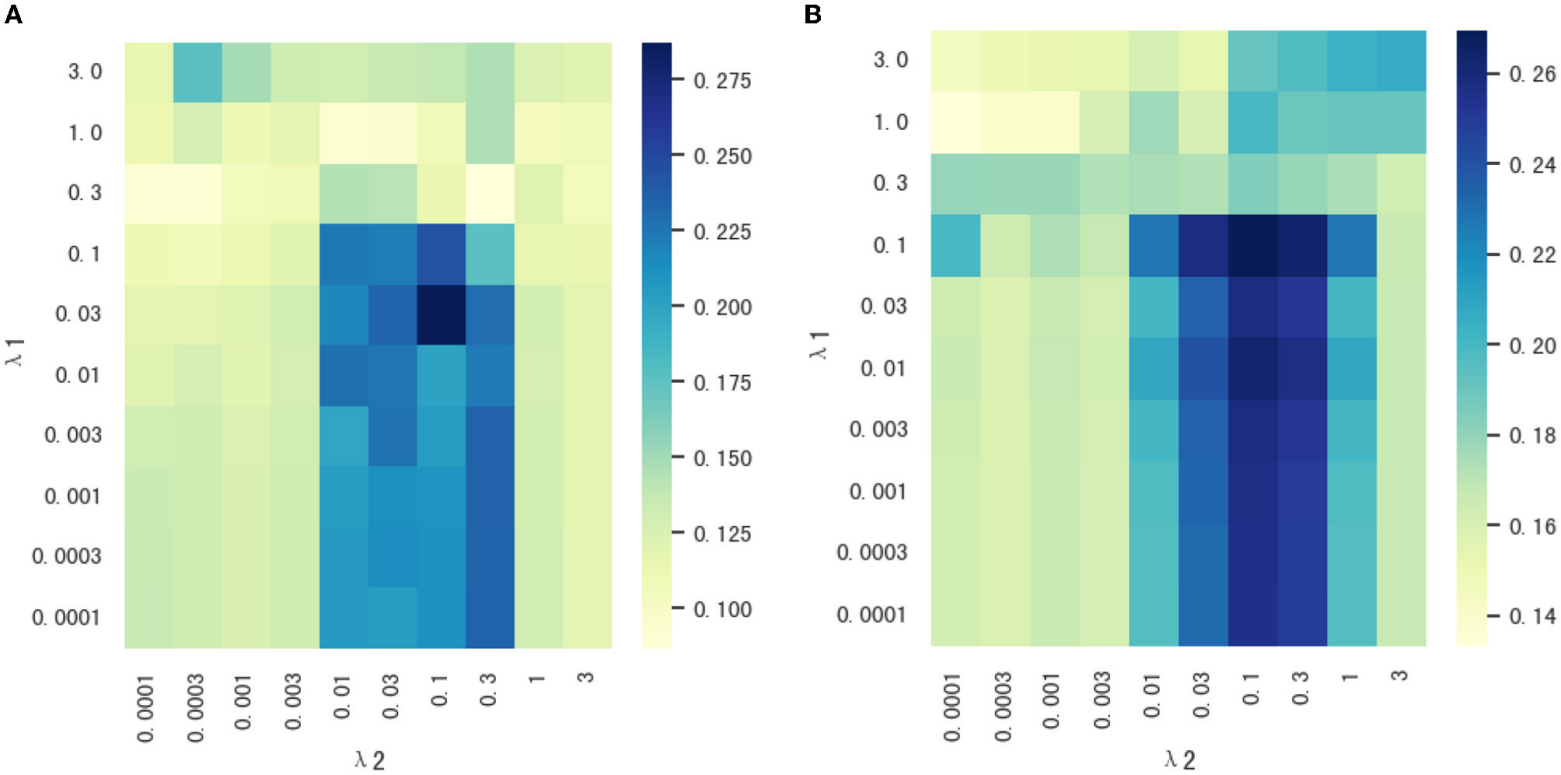
**(A, B)** The heat maps of correlation coefficients between parameters λ_1_ and λ_2_ on node and edge features.

Our proposed method, MSD-MM, contains two regularization parameters, the sparsity parameter λ_1_ and the multi-stage diagnosis information parameter λ_2_. The two parameters was used to balance the relative contributions of three terms in Equation (5). In order to research the effect of two regularization terms on the performance of our proposed method, we set the values of two parameters in the range of 10^−4^, 3 × 10^−4^, 10^−3^, 3 × 10^−3^, ..., 1, 3, respectively. Figure 5 shows the heat maps of correlation coefficients between parameters λ_1_ and λ_2_ on the test data. As shown in Figure 5, the MSD-MM method achieved the competitive or better performance than the MM method (reported in Table 3) on all combinations of parameter values, which further indicates the advantages of the multi-stage diagnosis information regularization term. Meanwhile, the areas were bounded by λ_1_ < 0.1 and 0.01 < λ_2_ < 0.3 consistently and obviously outperformed than the MM method on node and edge futures. This area was helpful to quickly select the optimal the values of two parameters in future research.

## 6. Conclusion

In summary, this study developed a novel imaging genetic association framework to mine the multi-modality phenotype network between a single genetic risk SNP and multi-stage diagnosis status. First, the multimodality phenotype network is constructed by the voxel node features and connectivity edge features from sMRI and rs-fMRI, respectively. After that, an association model incorporated multi-stage diagnosis status is used to fully explore the relationship between the MDD-risk SNP TPH1 rs1799913 and the multi-modality phenotype network. All participants were recruited from two hospitals, and each participant contains sMRI, rs-fMRI, and genotype data. The detailed experimental results show that our proposed method can improve the performance on the metrics of root mean squared error and correlation coefficient compared with other comparisons. Some consistent and stable ROIs biomarkers are identified from voxel node features and connectivity edge features of multi-modality phenotype network. Moreover, an interesting finding is that four new and risk SNPs were discovered highly associated with MDD.

This study is an initial attempt to explore the relationship between a single genetic MDD-risk SNP and multi-modality brain neuroimaging data (sMRI and rs-fMRI). In future, we further investigate the use of other modality brain imaging (e.g., DTI) to directly construct the multi-modality graphical phenotype network and mine relationship between multi-modality phenotype network and multi-locus risk SNPs. We hope that more meaningful results are discovered to deeper understand the pathogenesis of MDD and help the diagnosis and treatment of patients with MDD.

## Data availability statement

The original contributions presented in the study are publicly available. This data can be found here: https://doi.org/10.6084/m9.figshare.22015541.v1.

## Author contributions

LZ, YY, and DZ conceived and designed this article. LZ and MP proposed the association model, performed the experimental analysis, created figures and tables, and wrote this manuscripts. XL, CX, and ZZ collected the data set and organized it for analyses. MP trained the model and analyzed the experimental results with the help of XH and MW. All authors read, edited, and discussed the article. All authors contributed to the article and approved the submitted version.
